# Identifying Early Target Cells of Nipah Virus Infection in Syrian Hamsters

**DOI:** 10.1371/journal.pntd.0005120

**Published:** 2016-11-03

**Authors:** Laura Baseler, Dana P. Scott, Greg Saturday, Eva Horne, Rebecca Rosenke, Tina Thomas, Kimberly Meade-White, Elaine Haddock, Heinz Feldmann, Emmie de Wit

**Affiliations:** 1 Laboratory of Virology, National Institute of Allergy and Infectious Diseases, National Institutes of Health, Hamilton, Montana, United States of America; 2 Department of Comparative Pathobiology, Purdue University, West Lafayette, Indiana, United States of America; 3 Rocky Mountain Veterinary Branch, National Institute of Allergy and Infectious Diseases, National Institutes of Health, Hamilton, Montana, United States of America; University of Texas Medical Branch, UNITED STATES

## Abstract

**Background:**

Nipah virus causes respiratory and neurologic disease with case fatality rates up to 100% in individual outbreaks. End stage lesions have been described in the respiratory and nervous systems, vasculature and often lymphoid organs in fatal human cases; however, the initial target organs of Nipah virus infection have not been identified. Here, we detected the initial target tissues and cells of Nipah virus and tracked virus dissemination during the early phase of infection in Syrian hamsters inoculated with a Nipah virus isolate from Malaysia (NiV-M) or Bangladesh (NiV-B).

**Methodology/Principal Findings:**

Syrian hamsters were euthanized between 4 and 48 hours post intranasal inoculation and tissues were collected and analyzed for the presence of viral RNA, viral antigen and infectious virus.

Virus replication was first detected at 8 hours post inoculation (hpi). Nipah virus initially targeted type I pneumocytes, bronchiolar respiratory epithelium and alveolar macrophages in the lung and respiratory and olfactory epithelium lining the nasal turbinates. By 16 hpi, virus disseminated to epithelial cells lining the larynx and trachea. Although the pattern of viral dissemination was similar for both virus isolates, the rate of spread was slower for NiV-B. Infectious virus was not detected in the nervous system or blood and widespread vascular infection and lesions within lymphoid organs were not observed, even at 48 hpi.

**Conclusions/Significance:**

Nipah virus initially targets the respiratory system. Virus replication in the brain and infection of blood vessels in non-respiratory tissues does not occur during the early phase of infection. However, virus replicates early in olfactory epithelium and may serve as the first step towards nervous system dissemination, suggesting that development of vaccines that block virus dissemination or treatments that can access the brain and spinal cord and directly inhibit virus replication may be necessary for preventing central nervous system pathology.

## Author Summary

Nipah virus is a highly fatal paramyxovirus that causes respiratory and neurologic disease with widespread vascular damage. Although end stage disease has been characterized in humans and in animal models, the early phase of infection has yet to be described. Thus, it is known where the virus replicates during the final stages of infection, but not how the virus is transported to these end stage tissues or which tissues are targeted first. Using Syrian hamsters that were intranasally inoculated with virus as a model of Nipah virus infection, we show here that Nipah virus initially targets the respiratory tract, specifically the nasal cavity and lung, with virus replication being detected as early as 8 hours after inoculation. During the first 48 hours of infection, the virus slowly penetrates into the underlying tissues of the respiratory tract, but viremia and virus replication in the brain are not detected. In the lung, Nipah virus initially infects epithelial cells lining airways, followed by spread to arterial smooth muscle cells, suggesting that localized spread of the virus within an organ can lead to viral dissemination into the vasculature. Likewise, replication in the olfactory epithelium likely precedes spread of Nipah virus along the olfactory nerve into the brain. Understanding how Nipah virus spreads through the host to cause encephalitis is important for developing effective countermeasures that can target Nipah virus replication and prevent viral dissemination.

## Introduction

Nipah virus, a highly virulent paramyxovirus, was first identified in an outbreak in Malaysia and Singapore in 1998 and has since caused almost yearly outbreaks in humans in Bangladesh [1–6]. Nipah virus causes encephalitis and respiratory disease; however, the prevalence of respiratory symptoms and case fatality rates have varied among the reported outbreaks. Humans infected during the initial outbreak in Malaysia mainly exhibited neurologic symptoms while some also developed respiratory disease [7–10]. Outbreaks in Bangladesh have also resulted in neurologic disease, yet the incidence of respiratory disease has been higher, as have the case fatality rates [3, 11–13]. A definitive cause for the reported differences between these outbreaks has not yet been determined.

The pathology of Nipah virus in humans has only been described in fatal cases from the Malaysia outbreak; lesions were noted in the central nervous system, lung, vasculature and to a lesser extent the spleen and lymph nodes [1, 7, 9]. Nipah virus has been shown to affect the nervous, respiratory, vascular and immune systems, to varying degrees, by the end stage of disease in both humans and experimentally inoculated animals, including Syrian hamsters, ferrets, cats, guinea pigs and African green monkeys. Animal models have shown that by the late stages of disease, Nipah virus generally infects epithelial cells in the upper and lower respiratory tracts, endothelium and smooth muscle cells in arteries, neurons and mononuclear leukocytes regardless of inoculation route [14–20]. Although the end stage of Nipah virus disease is well characterized, the exact sequence and mechanism of virus dissemination through these different organ systems and cell types remains poorly defined for Nipah virus isolates from both Malaysia (NiV-M) and Bangladesh (NiV-B). Identifying the initial target organs and target cell types is vital to understanding the pathogenesis of Nipah virus and in potentially preventing virus dissemination.

Here, we identified the initial target tissues and cell types for Nipah virus during the first 48 hours post intranasal inoculation of Syrian hamsters. Syrian hamsters were used to model Nipah virus infection since they develop late stage lesions similar to those in humans [19–25]. Although both intranasal and intraperitoneal routes of Nipah virus inoculation have been described in Syrian hamsters, the intranasal route was chosen for this study since it more closely represents a potential natural route of Nipah virus infection in humans. In the Syrian hamsters, we showed that the lung and nasal turbinates were the initial target organs and that Nipah virus replication could be identified by 8 hours post inoculation (hpi) in type I pneumocytes, bronchiolar respiratory epithelial cells and alveolar macrophages and by 16 hpi in the nasal cavity respiratory and olfactory epithelial cells. Virus then appeared to disseminate to the trachea and larynx. We did not detect infectious virus or virus replication in the central nervous system or peripheral blood and there was no evidence of widespread viral infection of blood vessels, even at 48 hpi. These results suggest that Nipah virus first targets the respiratory system and that widespread virus dissemination and vascular and nervous system infection occur later.

## Methods

### Ethics and Biosafety Statements

All animal experiments were approved by the Institutional Animal Care and Use Committee of the Rocky Mountain Laboratories (RML; ASP#2014-088-E)) and carried out by certified staff in an Association for Assessment and Accreditation of Laboratory Animal Care (AAALAC) International accredited facility, according to the institution’s guidelines for animal use, and followed the guidelines and basic principles in the United States Public Health Service Policy on Humane Care and Use of Laboratory Animals (available from http://grants.nih.gov/grants/olaw/references/PHSPolicyLabAnimals.pdf), and the Guide for the Care and Use of Laboratory Animals (available from https://grants.nih.gov/grants/olaw/Guide-for-the-Care-and-use-of-laboratory-animals.pdf). All infectious work with Nipah virus was performed in a biosafety level 4 (BSL4) laboratory in the Integrated Research Facility at RML. Sample inactivation and removal of samples from the BSL4 laboratory was performed according to established standard operating procedures [26] approved by the Institutional Biosafety Committee (IBC).

### Virus and Cells

Nipah virus isolates from Bangladesh and Malaysia were kindly provided by the Special Pathogens Branch of the Centers for Disease Control and Prevention, Atlanta, GA. NiV-B was isolated from a throat swab collected from a fatal human case in 2004 and had been passaged three times in VeroE6 cells. NiV-M was isolated from the cerebrum of a fatal human case in 1999 and had been passaged four times in VeroE6 cells.

### Animal Experiments

Two groups of twenty-four 6- to 8-week-old female Syrian hamsters (HsdHan:AURA; Harlan Laboratories, Haslett, MI) were intranasally inoculated with 5 x 10^6^ TCID_50_ (50% tissue culture infectious dose) of either NiV-B or NiV-M in a total volume of 80 μl (40 μl per nostril). All hamsters were evaluated daily for clinical signs of disease. Four hamsters from each group were euthanized at 4, 8, 16, 24, 32 and 48 hpi. A terminal heart blood sample was collected from each hamster before necropsy. The nasal cavity, larynx, trachea, lung, cervical lymph nodes, spleen, brain and spinal cord were collected for histologic and virologic analysis.

### Histology, Immunohistochemistry and In Situ Hybridization

Necropsies and tissue sampling were performed according to a standard protocol approved by the IBC. Tissues were fixed for a minimum of 7 days in 10% neutral-buffered formalin and embedded in paraffin. The sections through the nasal turbinates, which were contained within the nasal cavity, and the spinal cord, which was contained within the vertebrae, were decalcified using a 20% EDTA solution in sucrose prior to paraffin embedding.

Leukocytes were isolated from terminal blood samples using centrifugation over a histopaque gradient (Sigma-Aldrich, St. Louis, MO) in conjunction with erythrolysis using ACK lysing buffer (Thermo Fisher Scientific, Waltham, MA) according to manufacturer instructions. The resulting leukocyte pellets were fixed for a minimum of 24 hours in 10% neutral-buffered formalin. Leukocytes from hamsters inoculated with the same Nipah virus isolate and which were euthanized at the same time point were pooled, then processed in HistoGel (Thermo Fisher Scientific) according to manufacturer instructions and embedded in paraffin to form a cell block.

Routine hematoxylin and eosin (H&E) staining, immunohistochemistry (IHC) and *in situ* hybridization (ISH) were performed on tissue sections and cell blocks. Nipah virus antigen was detected by IHC; tissue sections were labeled with a rabbit polyclonal antiserum against Nipah virus nucleoprotein (1:5000; kindly provided by L. Wang, Duke-NUS Medical School, Singapore) [27]. Nipah virus replication was detected in tissue sections by ISH using probes specific for positive sense Nipah virus nucleoprotein RNA using a previously described method [28]. All slides were evaluated by a board certified veterinary anatomic pathologist.

### Quantitative Real-Time RT-PCR (qRT-PCR)

Viral RNA was isolated from hamster tissues using the RNeasy Mini kit (Qiagen, Valencia, CA) or from hamster blood using the QIAamp Viral RNA Mini kit (Qiagen), according to manufacturer instructions. 5 μl of RNA was used in a one-step real-time RT-PCR targeting the Nipah virus nucleoprotein, as described previously [25], using the QuantiFast kit (Qiagen) according to manufacturer instructions. In each run, standard dilutions of RNA extracted from a titered virus stock were run in parallel, to calculate TCID_50_ equivalents in the samples.

### Virus Titrations

Virus titrations were performed by end-point titration in Vero C1008 cells. Vero C1008 cells were inoculated with tenfold serial dilutions of tissue homogenates. One hour after inoculation of cells with tissue homogenates, the inoculum was removed and replaced with 200 μl DMEM (Sigma-Aldrich) supplemented with 2% fetal bovine serum (HyClone, Logan, UT), 1 mM L-glutamine (Lonza, Walkersville, MD), 50 U/ml penicillin and 50 μg/ml streptomycin (Thermo Fisher Scientific). Five days after inoculation with tissue homogenates from NiV-B inoculated hamsters and three days after inoculation with tissue homogenates from NiV-M inoculated hamsters, when full cytopathic effect (CPE) was reached in Vero cells, CPE was scored and the TCID_50_ was calculated from 4 replicates by the Spearman-Karber method [29].

## Results

### Nipah virus replication is detected from 8 hpi onwards

**Fig 1:**
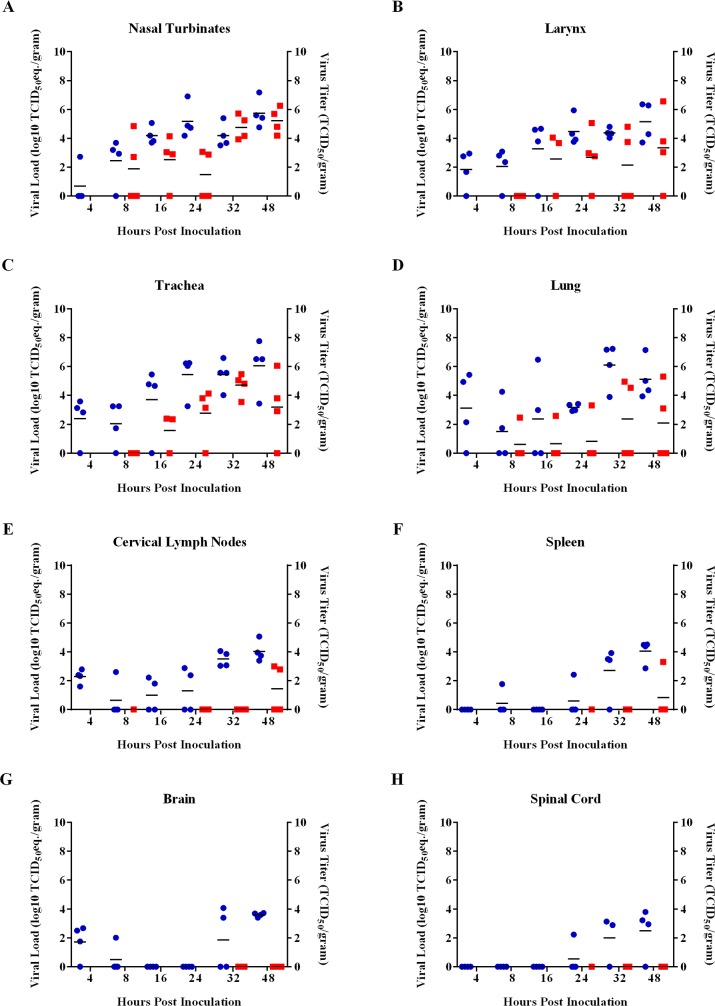
Viral loads and virus titers in respiratory, immune and nervous system tissues in hamsters inoculated with NiV-M. qRT-PCR was used to detect viral RNA and virus titration was used to detect infectious virus in the nasal turbinates (A), larynx (B), trachea (C), lung (D), cervical lymph nodes (E), spleen (F), brain (G) and spinal cord (H) at 4, 8, 16, 24, 32 and 48 hpi in Syrian hamsters intranasally inoculated with NiV-M. Viral loads in the tissues were determined as TCID_50_ equivalents. In each run, standard dilutions of RNA extracted from a titered virus stock were run in parallel, to calculate TCID_50_ equivalents. Virus titers in the tissues were determined by titration on Vero C1008 cells. Only samples that were taken at 8 hpi and onward and which were PCR positive were titered. Each dot indicates a single hamster; blue dots represent viral loads and red dots represent virus titers. Each horizontal line indicates the geometric mean viral load.

**Fig 2:**
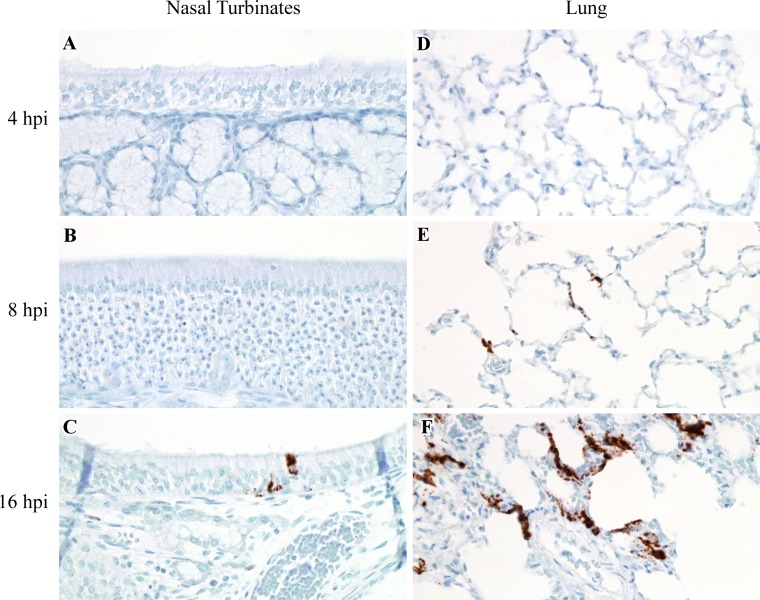
Virus replication in the nasal cavity and lung in hamsters inoculated with NiV-M. ISH was used to detect positive sense viral RNA, indicating virus replication, in the nasal cavity and lung at 4, 8 and 16 hpi in Syrian hamsters intranasally inoculated with NiV-M. Viral RNA is labeled brown in all images. Virus replication was not present in the nasal cavity at 4 or 8 hpi (A, B), but was observed at 16 hpi, as shown here in the respiratory epithelium lining a nasal turbinate (C). Virus replication was not identified at 4 hpi in the lung (D), yet was detected in pneumocytes at 8 and 16 hpi (E, F). All images were taken at 400x. A schematic representation of the hamster respiratory tract is shown in Fig 5.

To determine which tissues Nipah virus initially targets, we intranasally inoculated Syrian hamsters with 5 x 10^6^ TCID_50_ of NiV-M and analyzed tissues from the respiratory tract, central nervous system and immune system for the presence of Nipah virus RNA and infectious virus at multiple time points up to 48 hpi. Although viral RNA was detected by qRT-PCR in the lung, nasal turbinates, trachea, larynx, cervical lymph nodes and brain, but not the spleen and spinal cord at 4 hpi, infectious virus could be isolated only from the lung and nasal turbinates by 8 hpi, and from the trachea and larynx by 16 hpi (Fig 1). ISH using probes that targeted the positive sense RNA of the Nipah virus nucleoprotein, which is only observed when virus replication occurs, first detected virus replication at 8 hpi in the lung and 16 hpi in the nasal turbinates (Fig 2, Table 1). The lack of positive sense viral RNA at 4 hpi in all tissues examined, in addition to the decrease in detected mean viral loads in most tissues between 4 and 8 hpi, suggests that viral RNA detected at 4 hpi by qRT-PCR represented residual administered viral inoculum. Virus replication was not identified in the central nervous system or lymphoid organs at any of the time points tested. The absence of positive sense viral RNA and infectious virus from all tissues except the lung and nasal turbinates at 8 hpi suggests that the lung and nasal cavity were the initial target tissues for Nipah virus.

**Table 1:** Detection of virus replication by ISH in cells in the nasal cavity and lung of hamsters inoculated with NiV-M.

Cell Types	Time Post Inoculation (Hours)											
	4				8				16			
Nasal Cavity												
Respiratory epithelium	NE^1^	-	-	-	-	-	-	-	-	-	-	++
Olfactory epithelium	NE	NE	-	-	-	-	-	-	-	-	-	++
Submucosal gland epithelium	NE	NE	-	-	-	-	-	-	-	-	-	-
Lung												
Type I pneumocytes	-	-	-	-	++	-	++	-	+++	-	+++	-
Alveolar macrophages	-	-	-	-	++	-	++	-	+++	-	+++	-
Bronchiolar respiratory epithelium	-	-	-	-	+++	-	+++	-	+++	-	+++	-
Bronchiolar smooth muscle	-	-	-	-	-	-	-	-	-	-	-	-
Bronchial respiratory epithelium	-	-	-	-	-	-	-	-	++	-	+++	-
Arterial smooth muscle	-	-	-	-	-	-	-	-	-	-	-	-

Each column represents a single hamster.

The columns representing individual hamsters in this table correspond to the columns in Table 2.

The presence of positive sense Nipah virus RNA, indicating virus replication, was detected by ISH and was graded for individual cell types in the nasal cavity and lung.

Grading scale: -, negative; +, focal ISH signal; ++, multifocal mild ISH signal; +++, multifocal moderate ISH signal; ++++, multifocal to diffuse marked ISH signal.

^1^Not examined (NE). The cell type was not present in the tissue section examined.

### Nipah virus initially targets epithelial cells and macrophages

**Fig 3:**
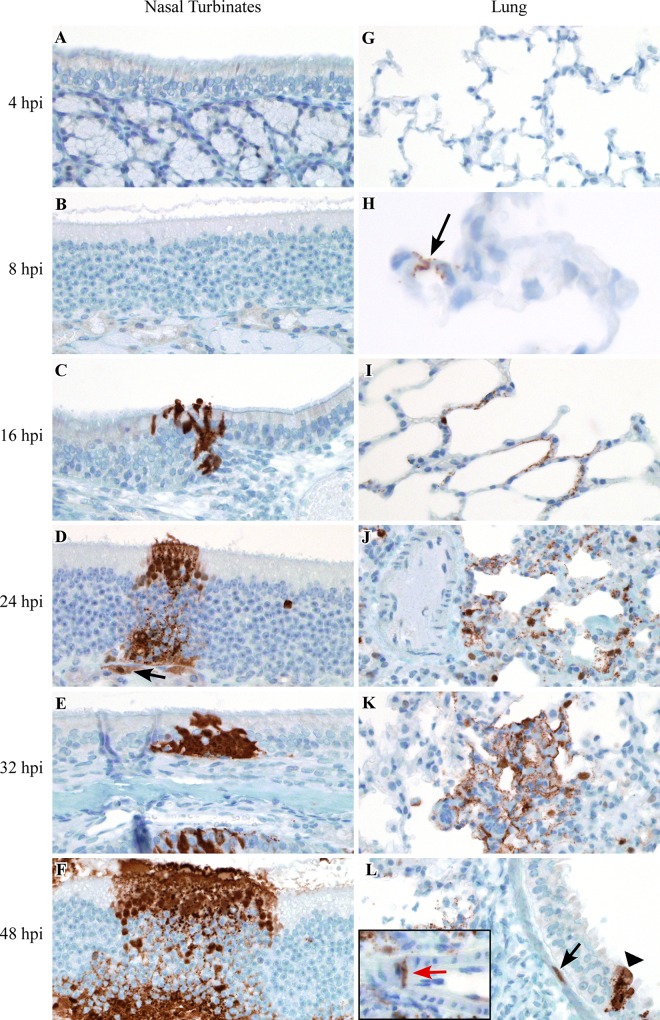
Dissemination of Nipah virus in the nasal cavity and lung of hamsters inoculated with NiV-M. IHC was used to detect the presence of viral antigen in the nasal cavity and lung at 4, 8, 16, 24, 32 and 48 hpi in Syrian hamsters intranasally inoculated with NiV-M. Viral antigen is labeled brown in all images. Viral antigen was not detected in the nasal cavity at 4 or 8 hpi (A, B). Viral antigen was first observed in the nasal cavity at 16 hpi, as shown here in respiratory epithelium lining a nasal turbinate (C). Increasing amounts of viral antigen were detected in olfactory and respiratory epithelium at subsequent time points (D-F). The spread of viral antigen to the submucosal glands was first detected at 24 hpi (arrow) (D). Nipah virus antigen was not observed at 4 hpi in the lung (G). Viral antigen was first detected at 8 hpi in the lung; arrow indicates antigen in type I pneumocytes (H). Increasing amounts of viral antigen were detected in pneumocytes at subsequent time points in the lung (I-L). Viral antigen was also detected in bronchiolar respiratory epithelium (arrowhead), bronchiolar smooth muscle (black arrow) and arterial smooth muscle (inset; red arrow) (L). The artery depicted in the inset (L) was from a different portion of the lung than that shown in the main image. All images were taken at 400x, except for (H) which was taken at 630x. A schematic representation of the hamster respiratory tract is shown in Fig 5.

To assess which cell types in the lung and nasal turbinates Nipah virus initially binds to and replicates in, we examined these tissues for the presence of positive sense viral RNA using ISH and Nipah virus nucleoprotein antigen using IHC. Positive sense viral RNA, indicating Nipah virus had bound to, infected and replicated in a cell, was first detected at 8 hpi in the lung, where it was observed in type I pneumocytes, bronchiolar respiratory epithelium and alveolar macrophages in 2 out of 4 hamsters (Table 1). As early as 8 hpi, the location of viral antigen on IHC labeled slides was often shown to correspond to foci of acute minimal bronchointerstitial pneumonia that were identified on H&E stained slides. IHC was used to track virus dissemination throughout the lung between 4 and 48 hpi; increasing amounts of viral antigen were detected at subsequent time points (Fig 3, Table 2). By 16 hpi, Nipah virus had disseminated from alveoli and bronchioles to larger airways and viral antigen and virus replication were observed in the bronchial respiratory epithelium in 2 out of 4 hamsters. Within bronchioles, viral antigen spread from the bronchiolar epithelium to the underlying bronchiolar smooth muscle in 1 out of 4 hamsters at 16 hpi. Dissemination of viral antigen to arterial smooth muscle cells in the lung was only noted at 32 and 48 hpi in 1 out of 4 hamsters and affected one to a few small-caliber arteries in areas exhibiting bronchointerstitial pneumonia; viral antigen was not observed in the endothelial cells of these vessels.

**Table 2:** Detection of viral antigen by IHC in cells in the nasal cavity and lung of hamsters inoculated with NiV-M.

Cell Types	Time Post Inoculation (Hours)																							
	4				8				16				24				32				48			
Nasal Cavity																								
Respiratory epithelium	-	-	-	-	-	-	-	-	+	-	-	+	+	-	NE^1^	++	++	+	+	-	++	++	++	+
Olfactory epithelium	-	-	-	-	-	-	-	-	-	-	-	++	++	-	NE	++	++	++	-	-	+++	-	++++	+
Submucosal gland epithelium	-	-	-	-	-	-	-	-	-	-	-	-	-	-	NE	++	++	-	-	-	++	-	+++	-
Lung																								
Type I pneumocytes	-	-	-	-	++	-	++	-	++	-	+++	-	-	++	-	-	-	+++	-	+++	++++	-	++	-
Alveolar macrophages	-	-	-	-	++	-	++	-	++	-	+++	-	-	++	-	-	+	+++	-	+++	++++	-	++	-
Bronchiolar respiratory epithelium	-	-	-	-	+	-	+++	-	++	-	+++	-	-	+++	-	-	++	+++	-	+++	+++	-	++	-
Bronchiolar smooth muscle	-	-	-	-	-	-	-	-	-	-	+	-	-	-	-	-	-	-	-	-	++	-	-	-
Bronchial respiratory epithelium	-	-	-	-	-	-	-	-	++	-	++	-	-	+++	-	-	++	++	-	+++	+++	-	+++	-
Arterial smooth muscle	-	-	-	-	-	-	-	-	-	-	-	-	-	-	-	-	-	-	-	++	+	-	-	-

Each column represents a single hamster.

The columns representing individual hamsters in this table correspond to the columns in Table 1.

The presence of Nipah virus antigen, as detected by IHC, was graded in cell types in the nasal cavity and lung.

Grading scale: -, negative; +, focal immunopositivity; ++, multifocal mild immunopositivity; +++, multifocal moderate immunopositivity; ++++, multifocal to diffuse marked immunopositivity.

^1^Not examined (NE). The cell type was not present in the tissue section examined.

In the nasal cavity, virus replication was first observed in respiratory and olfactory epithelium lining the nasal turbinates at 16 hpi (Table 1). Tracking of viral antigen in the nasal cavity over time showed that respiratory and olfactory epithelium were infected first, followed by the submucosal gland epithelium underlying both respiratory and olfactory epithelial cells at 24 hpi (Fig 3, Table 2). A mild neutrophilic rhinitis was identified on H&E slides as early as 16 hpi. Foci of rhinitis were often observed in locations where virus antigen was detected by IHC. Viral antigen was not observed in the vascular smooth muscle cells or endothelium in the nasal turbinates at any time point.

### Nipah virus spreads from the nasal cavity or lung to the trachea and larynx

**Fig 4:**
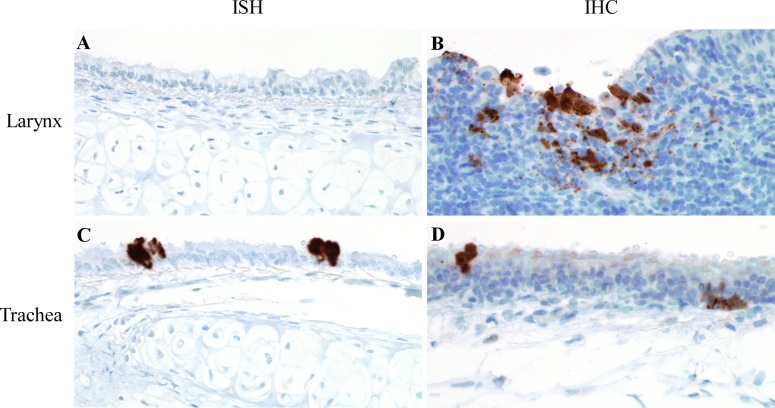
Virus replication and viral antigen in larynx and trachea of hamsters inoculated with NiV-M. Positive sense viral RNA, indicating virus replication, was detected by ISH, while IHC was used to detect viral antigen in Syrian hamsters intranasally inoculated with NiV-M. Viral RNA and viral antigen are labeled brown in all images. Virus replication was not identified in the larynx in any hamster (A); however, viral antigen was detected in the larynx (B). Virus replication (C) and viral antigen (D) were identified in respiratory epithelial cells in the trachea. All images were taken at 400x. A schematic representation of the hamster respiratory tract is shown in Fig 5.

Although infectious virus was detected at 8 hpi in the nasal cavity and lung of hamsters intranasally inoculated with NiV-M, it was not until later time points that Nipah virus was detected in the trachea and larynx. At 16 hpi, positive sense viral RNA, as analyzed by ISH, and Nipah virus antigen, detected by IHC, were identified in the trachea in 2 out of 4 hamsters (Fig 4). Infectious virus was isolated from both the trachea and larynx by 16 hpi (Fig 1). However, viral antigen was first detected in the larynx at 32 hpi and positive sense viral RNA was not identified in this tissue at any time point examined (Fig 4) despite detection of infectious virus and viral RNA, by qRT-PCR (Fig 1). As ISH and IHC are evaluated histologically, it is possible that positive sense viral RNA and viral antigen may have also been present in the larynx at 16 hpi, but were not found in the exact tissue sections that were labeled and examined. The lack of virus replication, infectious virus and viral RNA at 8 hpi in both of these tissues suggests that Nipah virus does not initially target the trachea and larynx. When viral antigen and virus replication were present in the trachea and larynx, they were identified in epithelial cells lining these airways and were not observed in blood vessels, suggesting that Nipah virus secondarily spreads from one or both of its initial target tissues, the nasal cavity and lung, to the trachea and larynx through the airways. Replication of virus in the nasal cavity and lung leads to increasing virus titers in these tissues and potentially increased numbers of virus particles disseminating to the larynx and trachea.

### Nipah virus dissemination outside of the respiratory tract

Since lesions have occasionally been identified in the spleen and lymph nodes of fatal human cases of Nipah virus infection [7], we examined these lymphoid organs for the presence of infectious virus, viral RNA and viral antigen in hamsters. Although viral RNA could be detected by qRT-PCR in cervical lymph nodes of hamsters from 4 hpi onwards, infectious virus was only detected at 48 hpi in the cervical lymph nodes of 2 out of 4 hamsters (Fig 1). Viral RNA was detected sporadically in the spleen at early time points and infectious virus was detected in the spleen of 1 out of 4 hamsters at 48 hpi (Fig 1). Positive sense viral RNA and viral antigen were not observed in the lymphoid organs at any time point.

To determine if Nipah virus had disseminated to the central nervous system, we analyzed both the brain and spinal cord of NiV-M inoculated hamsters for the presence of viral RNA, infectious virus and viral antigen. Low viral loads were detected by qRT-PCR in the brain and spinal cord of a few hamsters, with an increase in mean viral loads between 32 and 48 hpi (Fig 1). However, neither infectious virus (Fig 1), viral antigen, nor positive sense viral RNA were identified in the central nervous system tissues at any time point.

**Fig 5:**
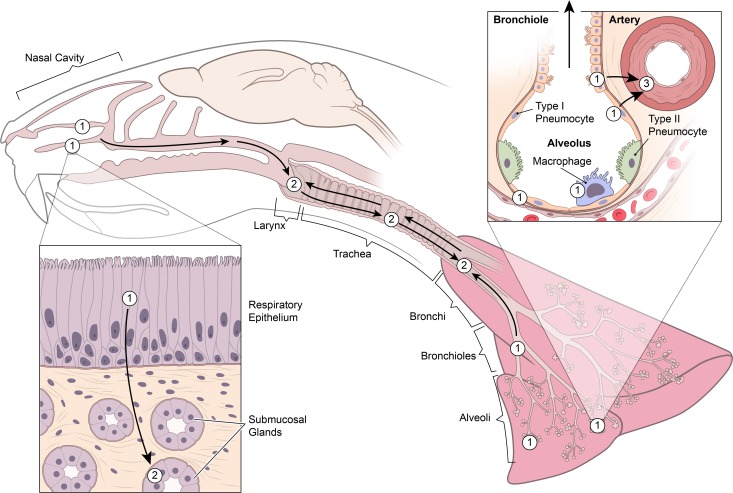
Schematic representation of the early dissemination of NiV through the upper and lower respiratory tract. Nipah virus initially infects respiratory and olfactory epithelium lining the nasal turbinates and type I pneumocytes, bronchiolar respiratory epithelium and alveolar macrophages in the lung (1). Nipah virus then spreads from the epithelium in the nasal cavity to the underlying submucosal glands (2). At the same time, the virus also moves downward from the nasal cavity and/or upward from the lung to infect epithelial cells lining the larynx, trachea and bronchi (2). Subsequently, Nipah virus infects the smooth muscle of arterial walls in the lung, likely as a result of local spread from adjacent infected pneumocytes and bronchiolar respiratory epithelial cells (3). Of note, NiV-M and NiV-B exhibit a similar dissemination pattern; however, the rate of spread is slower with NiV-B.

It has been shown that Nipah virus can bind to human lymphocytes and hamster mononuclear leukocytes which may then transfer the virus to permissive cells, such as endothelial cells, thereby potentially resulting in systemic viral dissemination [30]. To determine if Nipah virus binds to or replicates in circulating leukocytes during the early stage of infection, we performed IHC and ISH on cell blocks made up of leukocytes collected during terminal heart bleeds. Viral antigen and positive sense viral RNA were not observed in leukocytes in the peripheral blood at any time point. Additionally, viral RNA was not detected in the peripheral blood by qRT-PCR. These results suggest that Nipah virus either did not bind to, or replicate in, leukocytes circulating in blood or the level of virus in the blood was below the limit of detection. Furthermore, even at 48 hpi, viral antigen and positive sense viral RNA were not identified in the vasculature of any organ examined, other than the lung. Additionally, lesions associated with Nipah virus infection of blood vessels, including vasculitis, fibrinoid change and fibrin thrombi, were not detected histologically at any time point in any organ, except the lung. In the lung, viral antigen was only detected in the vascular wall of blood vessels located in foci of bronchointerstitial pneumonia. This suggests that the virus first has to infect and replicate in superficial cells lining the respiratory tract before the virus infiltrates deeper structures such as blood vessels (Fig 5), where it appears to invade from the outer vascular wall towards the endothelium and then finally enter the bloodstream, which may explain why viremia was still below the detection limit at 48 hpi.

### NiV-B mirrors the early targets of NiV-M yet exhibits slower virus dissemination within the nasal cavity and lung

Since the human case fatality rates and prevalence of respiratory disease were different between Nipah virus outbreaks in Malaysia and Bangladesh [8, 10, 12, 13], we sought to determine whether there were differences in the early pathogenesis of NiV-M and NiV-B infections.

Similar to hamsters inoculated with NiV-M, viral RNA was detected by qRT-PCR in the respiratory and central nervous systems and cervical lymph nodes at 4 hpi in hamsters intranasally inoculated with NiV-B (S1 Fig). Despite the detection of viral RNA in tissues from multiple organ systems at 4 hpi, positive sense viral RNA, indicative of virus replication, was only detected in the nasal turbinates and lung from 8 hpi onwards (S1 Fig and S2 Fig). Virus replication was observed at 8 hpi in type I pneumocytes and alveolar macrophages in 4 out of 4 hamsters and in bronchiolar respiratory epithelium in 2 out of 4 hamsters (S1 Fig, S2 Fig and Fig 5). Similar to NiV-M inoculated hamsters, the respiratory and olfactory epithelium in the nasal turbinates were also early targets for virus replication (S1 Table, S2 Fig and Fig 5). The presence of Nipah virus antigen in the nasal turbinates and lung, as detected by IHC, mirrored what was detected by ISH (S2 Table, S3 Fig). Unlike NiV-M inoculated hamsters, dissemination of viral antigen to pulmonary bronchiolar and arterial smooth muscle cells was not observed, even at 48 hpi. Spread of viral antigen in the nasal cavity from the respiratory and olfactory epithelial cells to the underlying submucosal gland epithelial cells took longer in NiV-B inoculated hamsters and was not detected until 48 hpi, compared to 24 hpi in NiV-M inoculated hamsters. Similar to NiV-M, NiV-B appeared to spread from the nasal cavity or lung to the trachea and larynx (S4 Fig and Fig 5). Widespread vascular dissemination was not detected, nor was infectious virus, viral antigen or virus replication identified in the brain, spinal cord or lymphoid organs at any time point in NiV-B inoculated hamsters. Additionally, viral antigen, virus replication and viral RNA were not detected in peripheral blood leukocytes at any time point.

## Discussion

In this study, we identified the early target tissues and cells of Nipah virus and evaluated viral dissemination during the early stages of infection for both NiV-M and NiV-B in intranasally inoculated Syrian hamsters. Previous Nipah virus studies in Syrian hamsters have utilized either an intranasal or intraperitoneal route of inoculation and have shown that end stage lesions in Syrian hamsters inoculated by either route are similar [21, 22], suggesting that organ tropism is not affected by inoculation route. Moreover, the intranasal inoculation route likely represents a more natural route of inoculation for humans, as compared to intraperitoneal inoculation. In our study, the nasal cavity and lung were the initial target tissues for both virus isolates. Within these tissues, Nipah virus initially exhibited epitheliotropism and, in the lung, a predilection for alveolar macrophages. We showed that virus replication, as indicated by the presence of positive sense viral RNA, could be identified by 8 hpi in the lung in type I pneumocytes, bronchiolar respiratory epithelial cells and alveolar macrophages. The presence of positive sense viral RNA in alveolar macrophages suggests virus replication occurred early in this cell type; however, phagocytosis is a major function of alveolar macrophages and if virus replication in epithelial cells began between the 4 and 8 hpi time points, it cannot be ruled out that the positive sense viral RNA detected in alveolar macrophages simply represented phagocytosis of virus infected epithelial cells [31].

In the nasal cavity and lung, virus infection and replication were first identified in epithelial cells that lined air spaces. Once in the nasal cavity and lung, Nipah virus disseminated from superficial epithelial cells to adjacent underlying cells, including bronchiolar smooth muscle cells, arterial smooth muscle cells in the lung and submucosal gland epithelial cells in the nasal cavity. Interestingly, our results showed that in the early phase of infection the spread of Nipah virus from epithelial cells that lined airways in the lung and nasal cavity to underlying cells appeared to be faster in hamsters inoculated with NiV-M than with NiV-B, suggesting that development of severe disease may occur faster for NiV-M. However, in studies evaluating late stage Nipah virus disease in Syrian hamsters, results were contradictory as to whether disease progression in the respiratory system was faster for NiV-M or for NiV-B [20, 21]. Additionally, end stage lesions in the respiratory tract appeared to plateau at the same severity level by 4 days post inoculation in Syrian hamsters inoculated with either Nipah virus isolate [20]. Similar studies analyzing late stage disease have been performed in ferrets and African green monkeys. Inoculation of ferrets with either NiV-M or NiV-B resulted in comparable terminal lesions and clinical signs, despite differences in oral viral shedding and viremia [16], while in African green monkeys NiV-B appeared to replicate more quickly and end stage respiratory lesions were more severe in animals inoculated with NiV-B than NiV-M [32]. The results from the ferret and hamster models of Nipah virus suggest that differences in the rate of virus replication or dissemination noted prior to end stage disease may not in fact significantly impact the overall clinical disease severity or outcome, while the African green monkey model suggests otherwise and further evaluation of the early pathogenesis of Nipah virus in African green monkeys is needed to determine if differences exist between these models.

Although widespread viral infection of blood vessels with development of histologic lesions has been reported in fatal human cases [1, 7], this was not identified at any time point in the hamsters in this experiment, implying that widespread vascular involvement does not occur during the early phase of infection. In some of the later time points in NiV-M inoculated hamsters in our study, Nipah virus antigen was present in pneumocytes adjacent to multiple arteries in the lung and Nipah virus antigen was identified in the arterial tunica media, but not endothelial cells in these blood vessels. These results suggest that Nipah virus spread from the adjacent pulmonary parenchyma to the outer vascular wall of arteries, with successive dissemination towards the vascular lumen. If time points subsequent to 48 hpi were analyzed it is likely that Nipah virus would have spread from the arterial smooth muscle cells to the overlying endothelial cells in the lung, then into the bloodstream with subsequent infection of blood vessels throughout the body, as evidenced by detection of viral antigen in endothelial cells in multiple tissues in Syrian hamster studies focusing on the late stages of disease [19–22]. Although the exact route of systemic virus dissemination to tissues and vasculature throughout the body is still unclear, it is suspected that circulating lymphocytes may play a role. Even though we were not able to detect virus binding to, or infecting, peripheral blood leukocytes during the early stage of infection in hamsters, it has been shown that Nipah virus can bind to human lymphocytes and hamster mononuclear leukocytes and that these leukocytes can carry and transfer the virus to permissive cells [30]. As such, lymphocytes may transfer virus directly to endothelial cells in blood vessels throughout the body, with secondary viral spread from blood vessels to adjacent parenchymal cells, including epithelial cells and neurons, or transmigration of lymphocytes through vascular walls and direct transfer of the virus to parenchymal cells during the later stages of infection [33].

Subsequent to the initial infection of cells in the nasal cavity and lung, epithelial cells lining the larynx and trachea were targeted by Nipah virus. Based on the lack of vascular involvement at this time point, the dissemination of Nipah virus throughout the respiratory tract was likely caused by the upward or downward spread of virus particles through the airways during respiration, or from the bronchi to the trachea and larynx by way of the mucociliary apparatus rather than via a hematogenous route. Other viruses that target the respiratory tract, including influenza virus, have been shown to damage the mucociliary apparatus, thereby decreasing the speed and effectiveness with which pathogens are cleared from the respiratory tract [34]. Alternatively, the epithelium lining the larynx and trachea may have been exposed to Nipah virus at the same time point as the nasal cavity and lung, but the rate of viral entry and replication in the larynx and trachea may have been slower resulting in delayed detection of viral antigen and replication.

Since histologic lesions have been reported in the central nervous system, and to a lesser extent, lymphoid organs in fatal human cases [1, 7], we evaluated whether there was virus dissemination to non-respiratory tract tissues during the early stages of Nipah virus infection in Syrian hamsters. Low viral loads were observed in the brain and spinal cord in a few NiV-B and NiV-M inoculated hamsters at 24 or 32 hpi, yet infectious virus, viral antigen, and virus replication were not detected. The low viral loads in these tissues may signify early virus dissemination to the nervous system at a level below the detection limit of IHC and virus titrations. It has previously been shown that IHC can detect viral antigen in the brain of Syrian hamsters by 4 days post inoculation and that virus disseminated from the olfactory epithelium to the brain through the olfactory nerve [23]. Our results likely represent the initial stages of virus dissemination to the nervous system; however, since viral loads in the brain were low, we were unable to detect, or track movement of, viral antigen into the brain by IHC. Spread of virus from the brain to the spinal cord may have occurred through the cerebrospinal fluid. Nipah virus has been detected in the cerebrospinal fluid of infected humans [35]. In the lymphoid organs, infectious virus was detected at 48 hpi in the cervical lymph nodes of NiV-M inoculated hamsters, but was not present in NiV-B inoculated hamsters. Lymphatics from the head and neck drain into the cervical lymph nodes, as such, virus could be transported from the nasal cavity to the cervical lymph nodes through lymphatic drainage. Infectious virus was only detected at 48 hpi in the spleen of a single NiV-M inoculated hamster and was not identified in any NiV-B inoculated hamsters. Spread of Nipah virus to the spleen may have occurred by a hematogenous route. Although viremia was not detected at 48 hpi, it is possible that the overall viral load in the blood was below the level of detection; however, since the spleen functions to remove pathogens from the blood as blood is filtered through the spleen [36–38], Nipah virus may have accumulated to high enough levels in the spleen that virus could be detected there.

In summary, our results provide evidence that Nipah virus initially disseminates throughout the upper and lower respiratory tracts via airways during the early phase of infection after intranasal inoculation. This may be followed by dissemination of virus from the nasal cavity to the nervous system by neural route and dissemination of virus to lymph nodes via lymphatic drainage. Dissemination of virus to blood vessels outside of the respiratory tract does not appear to occur during the first 48 hpi and is considered a late stage event. Since Nipah virus can cause encephalitis that results in severe neurologic disease in humans and brainstem damage that often causes death, either by directly infecting neurons or infecting nervous system blood vessels with secondary development of infarcts, it is important to prevent virus dissemination and replication in the brain and vasculature [1, 5, 7, 9]. Our data indicate that virus replication and dissemination are initiated rapidly after viral infection, thus making it difficult to prevent the virus from spreading to non-respiratory tract tissues such as the central nervous system. This suggests that development of vaccines that block dissemination or treatments that can access the brain and spinal cord and directly inhibit viral entrance into cells and/or virus replication may be necessary for prevention of central nervous system pathology.

## Supporting Information

S1 Fig Viral loads and virus titers in respiratory, immune and nervous system tissues in hamsters inoculated with NiV-B. qRT-PCR was used to detect viral RNA and virus titration was used to detect infectious virus in the nasal turbinates (A), larynx (B), trachea (C), lung (D), cervical lymph nodes (E), spleen (F), brain (G) and spinal cord (H) at 4, 8, 16, 24, 32 and 48 hpi in Syrian hamsters intranasally inoculated with NiV-B. Viral loads in the tissues were determined as TCID_50_ equivalents. In each run, standard dilutions of RNA extracted from a titered virus stock were run in parallel, to calculate TCID_50_ equivalents. Virus titers in the tissues were determined by titration on Vero C1008 cells. Only samples that were taken at 8 hpi and onward and which were PCR positive were titered. Each dot indicates a single hamster; blue dots represent viral loads and red dots represent virus titers. Each horizontal line indicates the geometric mean viral load.
(TIF)

S2 Fig Virus replication in the nasal cavity and lung in hamsters inoculated with NiV-B. ISH was used to detect positive sense viral RNA, indicating virus replication, in the nasal cavity and lung at 4, 8 and 16 hpi in Syrian hamsters intranasally inoculated with NiV-B. Viral RNA is labeled brown in all images. Virus replication was not present in the nasal cavity at 4 or 8 hpi (A, B), yet was observed at 16 hpi, as shown here in the olfactory epithelium lining a nasal turbinate (C). Virus replication was not identified at 4 hpi in the lung (D), yet was detected in pneumocytes at 8 hpi (arrow) and 16 hpi (E, F). All images were taken at 400x.
(TIF)

S3 Fig Dissemination of Nipah virus in the nasal cavity and lung of hamsters inoculated with NiV-B. IHC was used to detect the presence of viral antigen in the nasal cavity and lung at 4, 8, 16, 24, 32 and 48 hpi in Syrian hamsters intranasally inoculated with NiV-B. Viral antigen is labeled brown in all images. Viral antigen was not detected in the nasal cavity at 4 or 8 hpi (A, B). Viral antigen was first observed in the nasal cavity at 16 hpi, as shown here in the olfactory epithelium lining a nasal turbinate (C). Increasing amounts of viral antigen were detected in olfactory and respiratory epithelium (D-F). The spread of viral antigen to the submucosal glands (arrow) was first detected at 48 hpi (F). Nipah virus antigen was not observed at 4 hpi in the lung (G). Viral antigen was first detected at 8 hpi in the lung; arrow indicates antigen in pneumocytes (H). Increasing amounts of viral antigen were typically detected in pneumocytes at subsequent time points in the lung (I-L). Viral antigen was also detected in bronchiolar respiratory epithelium (arrow) (K) and bronchial respiratory epithelium (L; inset). The inset in image L depicts an airway which is not visible in the main image. All images were taken at 400x, except for (H) which was taken at 630x.
(TIF)

S4 Fig Virus replication and viral antigen in larynx and trachea of hamsters inoculated with NiV-M. Positive sense viral RNA, indicating virus replication, was detected by ISH, while IHC was used to detect viral antigen in Syrian hamsters intranasally inoculated with NiV-B. Virus replication was identified in epithelial cells lining the larynx (A), although viral antigen was not detected (B). Virus replication was not detected in the trachea of any hamster (C); however, viral antigen was observed in epithelial cells lining the trachea (D). All images were taken at 400x.
(TIF)

S1 Table Detection of virus replication in cells in the nasal cavity and lung of hamsters inoculated with NiV-B. (DOCX)

S2 Table Detection of viral antigen in cells in the nasal cavity and lung of hamsters inoculated with NiV-B. (DOCX)
