# Supplementary figures and images for: Identifying Early Target Cells of Nipah Virus Infection in Syrian Hamsters
Source: PLoS Negl Trop Dis. 2016 Nov 3;10(11):e0005120. doi: 10.1371/journal.pntd.0005120 (PMC5094696; doi:10.1371/journal.pntd.0005120)

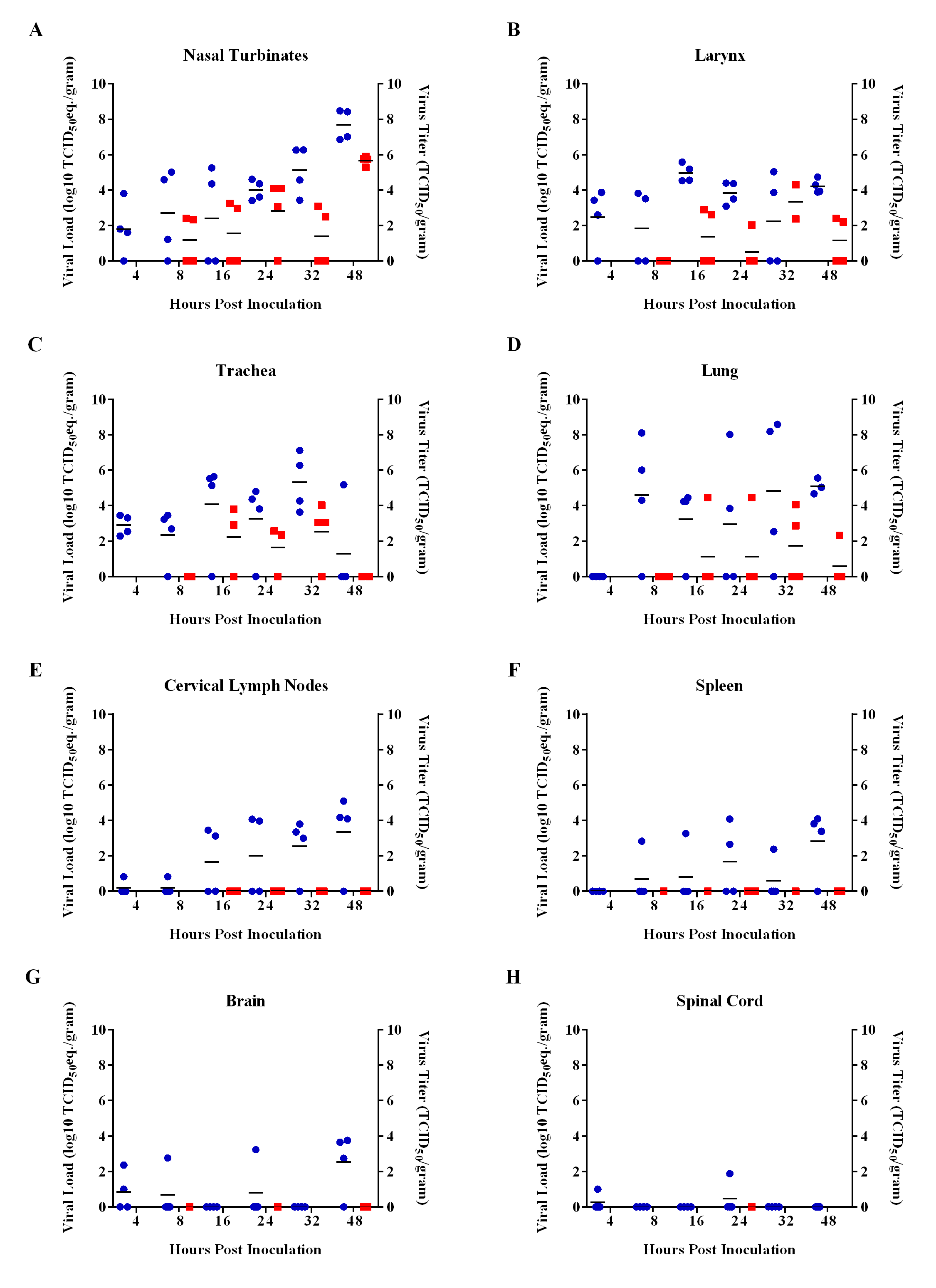

Supplement: S1 Fig — qRT-PCR was used to detect viral RNA and virus titration was used to detect infectious virus in the nasal turbinates (A), larynx (B), trachea (C), lung (D), cervical lymph nodes (E), spleen (F), brain (G) and spinal cord (H) at 4, 8, 16, 24, 32 and 48 hpi in Syrian hamsters intranasally inoculated with NiV-B. Viral loads in the tissues were determined as TCID50 equivalents. In each run, standard dilutions of RNA extracted from a titered virus stock were run in parallel, to calculate TCID50 equivalents. Virus titers in the tissues were determined by titration on Vero C1008 cells. Only samples that were taken at 8 hpi and onward and which were PCR positive were titered. Each dot indicates a single hamster; blue dots represent viral loads and red dots represent virus titers. Each horizontal line indicates the geometric mean viral load. (TIF) [file pntd.0005120.s001.tif]

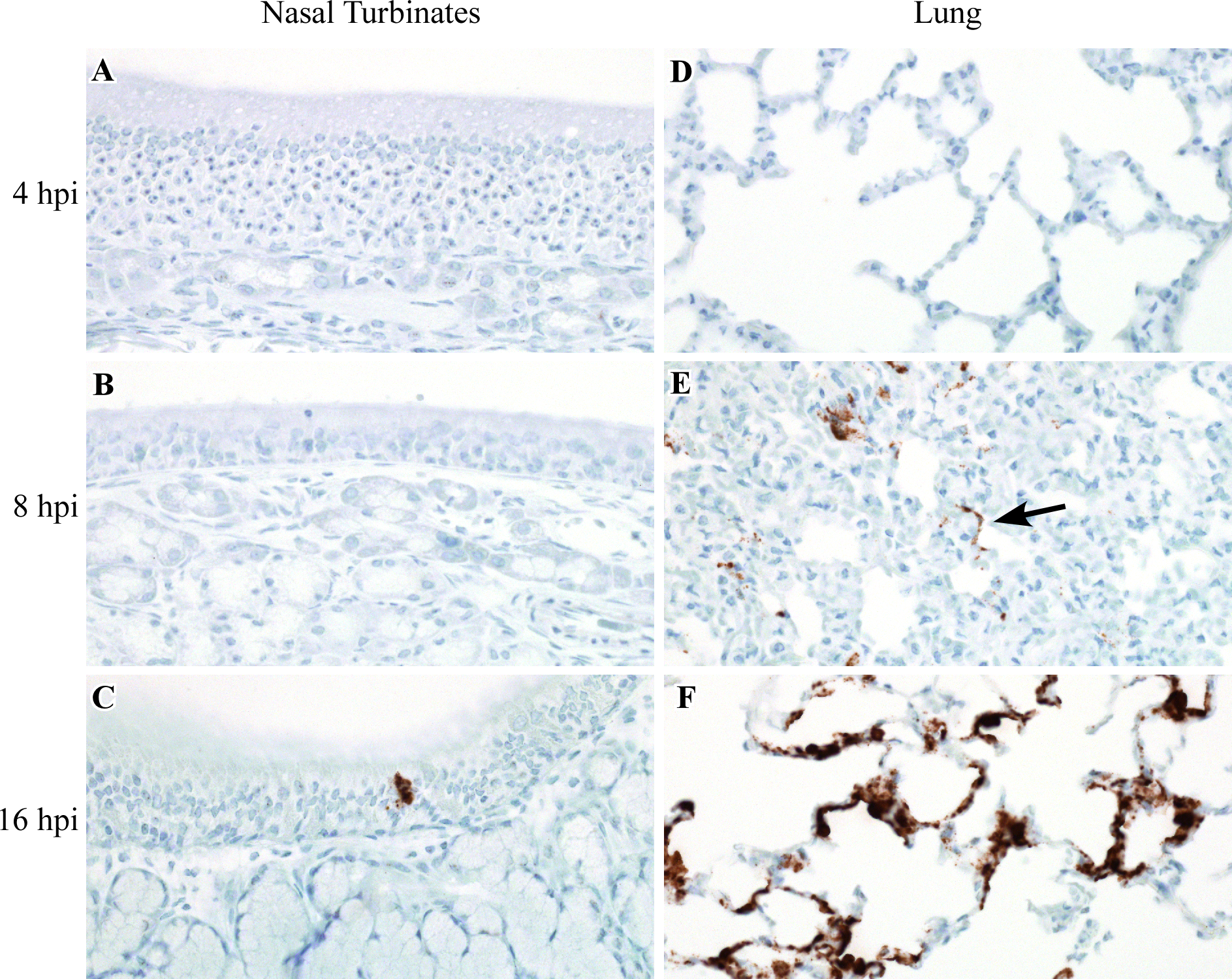

Supplement: S2 Fig — ISH was used to detect positive sense viral RNA, indicating virus replication, in the nasal cavity and lung at 4, 8 and 16 hpi in Syrian hamsters intranasally inoculated with NiV-B. Viral RNA is labeled brown in all images. Virus replication was not present in the nasal cavity at 4 or 8 hpi (A, B), yet was observed at 16 hpi, as shown here in the olfactory epithelium lining a nasal turbinate (C). Virus replication was not identified at 4 hpi in the lung (D), yet was detected in pneumocytes at 8 hpi (arrow) and 16 hpi (E, F). All images were taken at 400x. (TIF) [file pntd.0005120.s002.tif]

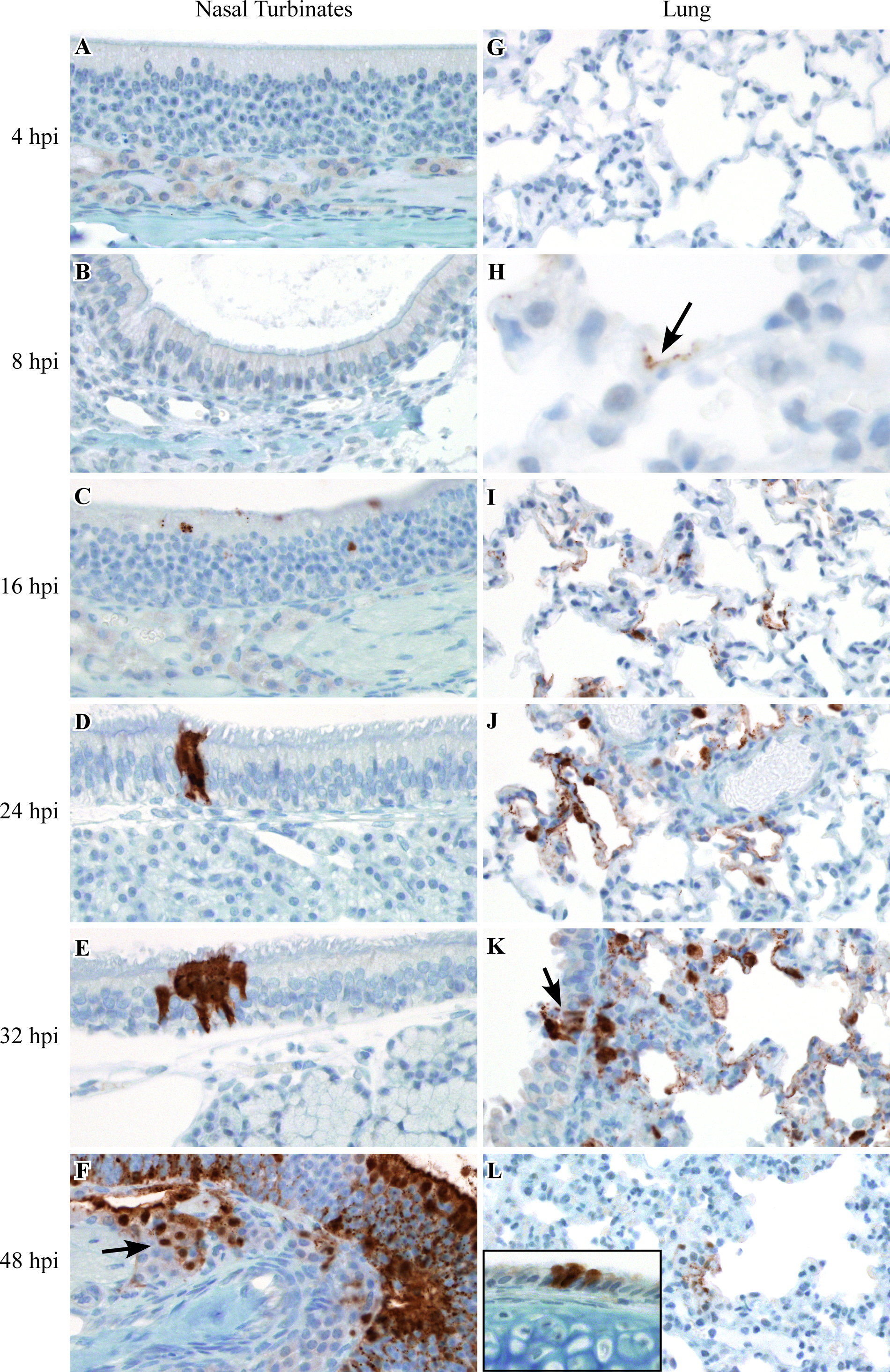

Supplement: S3 Fig — IHC was used to detect the presence of viral antigen in the nasal cavity and lung at 4, 8, 16, 24, 32 and 48 hpi in Syrian hamsters intranasally inoculated with NiV-B. Viral antigen is labeled brown in all images. Viral antigen was not detected in the nasal cavity at 4 or 8 hpi (A, B). Viral antigen was first observed in the nasal cavity at 16 hpi, as shown here in the olfactory epithelium lining a nasal turbinate (C). Increasing amounts of viral antigen were detected in olfactory and respiratory epithelium (D-F). The spread of viral antigen to the submucosal glands (arrow) was first detected at 48 hpi (F). Nipah virus antigen was not observed at 4 hpi in the lung (G). Viral antigen was first detected at 8 hpi in the lung; arrow indicates antigen in pneumocytes (H). Increasing amounts of viral antigen were typically detected in pneumocytes at subsequent time points in the lung (I-L). Viral antigen was also detected in bronchiolar respiratory epithelium (arrow) (K) and bronchial respiratory epithelium (L; inset). The inset in image L depicts an airway which is not visible in the main image. All images were taken at 400x, except for (H) which was taken at 630x. (TIF) [file pntd.0005120.s003.tif]

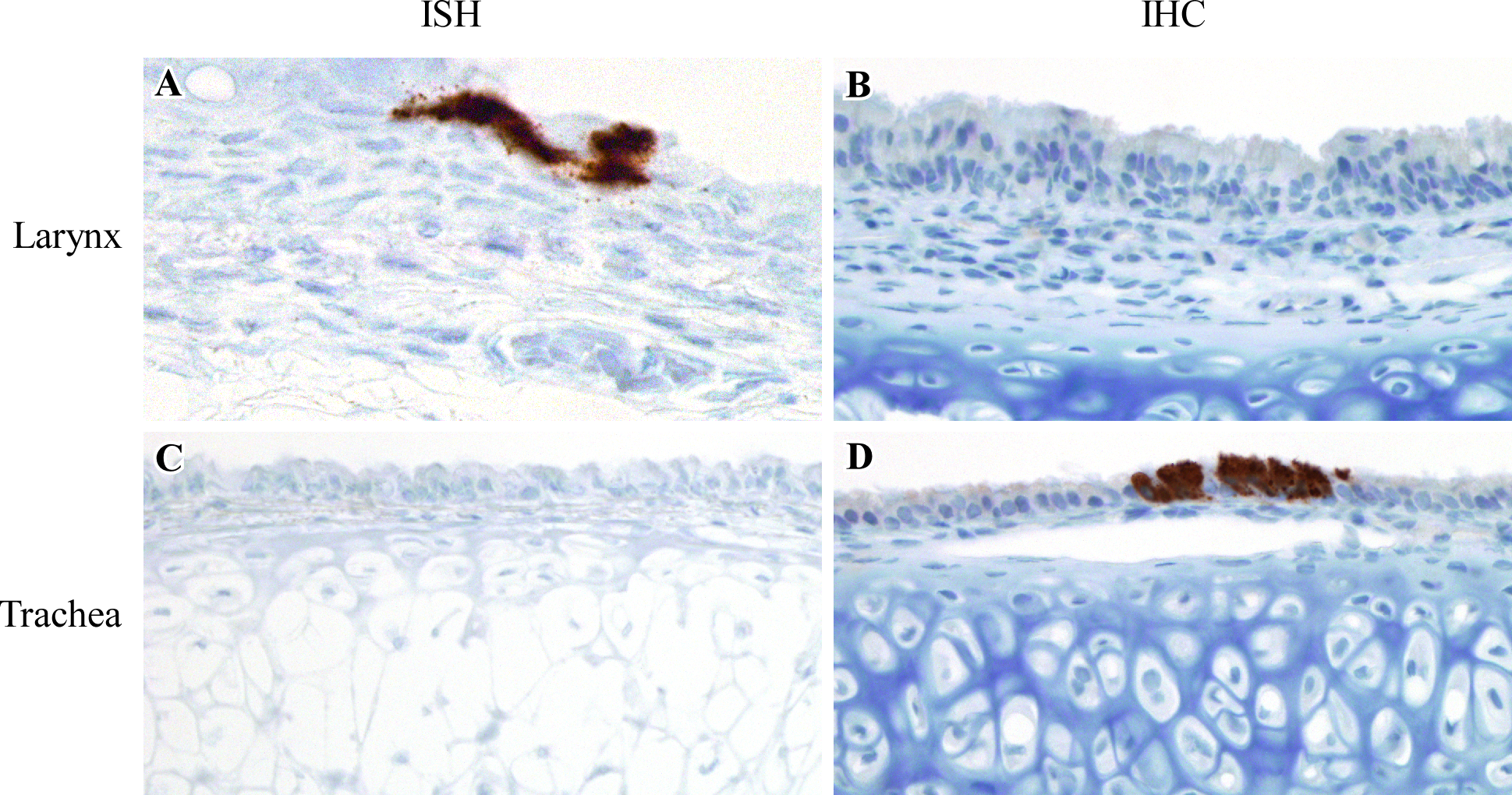

Supplement: S4 Fig — Positive sense viral RNA, indicating virus replication, was detected by ISH, while IHC was used to detect viral antigen in Syrian hamsters intranasally inoculated with NiV-B. Virus replication was identified in epithelial cells lining the larynx (A), although viral antigen was not detected (B). Virus replication was not detected in the trachea of any hamster (C); however, viral antigen was observed in epithelial cells lining the trachea (D). All images were taken at 400x. (TIF) [file pntd.0005120.s004.tif]
