# Supplementary material for: Identifying Early Target Cells of Nipah Virus Infection in Syrian Hamsters
Source: PLoS Negl Trop Dis. 2016 Nov 3;10(11):e0005120. doi: 10.1371/journal.pntd.0005120 (PMC5094696; doi:10.1371/journal.pntd.0005120)
Supplement: S1 Table — (DOCX) [file pntd.0005120.s005.docx]

**S1 Table. Detection of virus replication by in situ hybridization in cells in the nasal cavity of hamsters inoculated with NiV-B.**

| Cell Types | Time Post Inoculation (Hours) | | | | | | | | | | | | | | |
| --- | --- | --- | --- | --- | --- | --- | --- | --- | --- | --- | --- | --- | --- | --- | --- |
|  | 4 | | | | 8 | | | | | | | 16 | | | |
| Nasal Cavity |  |  |  |  |  |  | | |  | |  |  |  |  |  |
| Respiratory epithelium | - | - | - | - | - | | - | - | | - | | + | - | + | + |
| Olfactory epithelium | - | - | - | - | - | | - | - | | - | | ++ | ++ | - | - |
| Submucosal gland epithelium | - | - | - | - | - | | - | - | | - | | - | - | - | - |
| Lung |  |  |  |  |  | |  |  | |  | |  |  |  |  |
| Type I pneumocytes | - | - | - | - | ++ | | ++ | ++ | | ++ | | +++ | +++ | ++ | - |
| Alveolar macrophages | - | - | - | - | ++ | | ++ | ++ | | ++ | | +++ | +++ | ++ | - |
| Bronchiolar respiratory epithelium | - | - | - | - | + | | ++ | - | | - | | +++ | +++ | +++ | - |
| Bronchiolar smooth muscle | - | - | - | - | - | | - | - | | - | | - | - | - | - |
| Bronchial respiratory epithelium | - | - | - | - | ++ | | - | - | | - | | - | ++ | ++ | - |
| Arterial smooth muscle | - | - | - | - | - | | - | - | | - | | - | - | - | - |

Each column represents a single hamster. The columns representing individual hamsters in this table correspond to the columns in Table S2. The presence of positive sense Nipah virus RNA, indicating virus replication, was detected by ISH and was graded for individual cell types in the nasal cavity and lung. Grading scale: - , negative; +, focal ISH signal; ++, multifocal mild ISH signal; +++, multifocal moderate ISH signal; ++++, multifocal to diffuse marked ISH signal.
