# Supplementary material for: Identifying Early Target Cells of Nipah Virus Infection in Syrian Hamsters
Source: PLoS Negl Trop Dis. 2016 Nov 3;10(11):e0005120. doi: 10.1371/journal.pntd.0005120 (PMC5094696; doi:10.1371/journal.pntd.0005120)
Supplement: S2 Table — (DOCX) [file pntd.0005120.s006.docx]

**S2 Table. Detection of viral antigen by immunohistochemistry in cells in the nasal cavity and lung of hamsters inoculated with NiV-B.**

| Cell Types | Time Post Inoculation (Hours) | | | | | | | | | | | | | | | | | | | | | | | |
| --- | --- | --- | --- | --- | --- | --- | --- | --- | --- | --- | --- | --- | --- | --- | --- | --- | --- | --- | --- | --- | --- | --- | --- | --- |
|  | 4 | | | | 8 | | | | 16 | | | | 24 | | | | 32 | | | | 48 | | | |
| Nasal Cavity |  |  |  |  |  |  |  |  |  |  |  |  |  |  |  |  |  |  |  |  |  |  |  |  |
| Respiratory epithelium | - | - | - | - | - | - | - | - | ++ | - | + | - | - | ++ | + | - | ++ | - | + | - | ++ | - | ++ | + |
| Olfactory epithelium | - | - | - | - | - | - | - | - | ++ | + | - | - | - | - | - | - | ++ | - | + | - | - | - | +++ | - |
| Submucosal gland epithelium | - | - | - | - | - | - | - | - | - | - | - | - | - | - | - | - | - | - | - | - | - | - | +++ | - |
| Lung |  |  |  |  |  |  |  |  |  |  |  |  |  |  |  |  |  |  |  |  |  |  |  |  |
| Type I pneumocytes | - | - | - | - | ++ | - | ++ | ++ | +++ | +++ | ++ | - | - | - | ++ | ++ | ++ | +++ | - | +++ | + | - | ++ | + |
| Alveolar macrophages | - | - | - | - | ++ | - | ++ | ++ | +++ | +++ | ++ | - | - | - | ++ | ++ | ++ | +++ | - | +++ | - | - | + | + |
| Bronchiolar respiratory epithelium | - | - | - | - | + | - | - | + | ++ | ++ | + | - | - | - | - | + | + | +++ | - | +++ | + | - | - | - |
| Bronchiolar smooth muscle | - | - | - | - | - | - | - | - | - | - | - | - | - | - | - | - | - | - | - | - | - | - | - | - |
| Bronchial respiratory epithelium | - | - | - | - | - | - | - | + | - | - | - | - | - | - | - | - | - | + | - | - | - | - | - | ++ |
| Arterial smooth muscle | - | - | - | - | - | - | - | - | - | - | - | - | - | - | - | - | - | - | - | - | - | - | - | - |

Each column represents a single hamster. The columns representing individual hamsters in this table correspond to the columns in Table S1. The presence of Nipah virus antigen, as detected by IHC, was graded in cell types in the nasal cavity and lung. Grading scale: -, negative; +, focal immunopositivity; ++, multifocal mild immunopositivity; +++, multifocal moderate immunopositivity; ++++, multifocal to diffuse marked immunopositivity.
